# Successful thoracoscopic resection of costal osteochondroma causing pneumothorax

**DOI:** 10.1093/jscr/rjaf840

**Published:** 2025-10-22

**Authors:** Shogo Ide, Maho Seshimoto, Gaku Saito

**Affiliations:** Division of General Thoracic Surgery, National Hospital Organization Shinshu Ueda Medical Center, 1-27-21 Midorigaoka, Ueda, Nagano 386-8610, Japan; Division of General Thoracic Surgery, National Hospital Organization Shinshu Ueda Medical Center, 1-27-21 Midorigaoka, Ueda, Nagano 386-8610, Japan; Division of General Thoracic Surgery, National Hospital Organization Shinshu Ueda Medical Center, 1-27-21 Midorigaoka, Ueda, Nagano 386-8610, Japan

**Keywords:** pneumothorax, costal osteochondroma, thoracoscopic surgery, minimally invasive surgery, costal exostosis

## Abstract

Costal osteochondromas are rare and infrequently cause pneumothorax and/or hemothorax by injuring the intrathoracic structures. A 13-year-old boy presented with chest pain and dyspnea. Chest radiography revealed a left pneumothorax. Computed tomography revealed a bony and pedunculated mass arising from the fifth rib, pointing directly inward into the thoracic cavity. We suspected that the mass was a costal osteochondroma that had caused a pneumothorax by injuring the left upper lobe of the lungs. The patient underwent a thoracoscopic surgery. A costal osteochondroma arising from the fifth rib and a wound in the left lingular segment were found. We performed wedge resection of the left lingular segment and removed the osteochondroma. Histological examination revealed an osteochondroma with complete resection of the cartilage cap. The patient was discharged on postoperative day 4. We conducted postoperative observations and found no recurrence of the osteochondroma or pneumothorax.

## Introduction

Osteochondromas, also known as “exostosis,” are the most common bone tumors, accounting for 20%–50% of all benign bone tumors [1, 2]. Osteochondromas tend to occur at the metaphyseal region of the long bones of the extremities but have also been reported to exist in other areas, such as the scapula, pelvis, rib, and vertebra [1]. Osteochondromas occurring in the ribs account for 1%–2% of all cases [2]. Symptomatic osteochondromas arising from the rib are very rare. Herein, we report a case of a pneumothorax secondary to a costal osteochondroma.

## Case report

**Figure 1:**
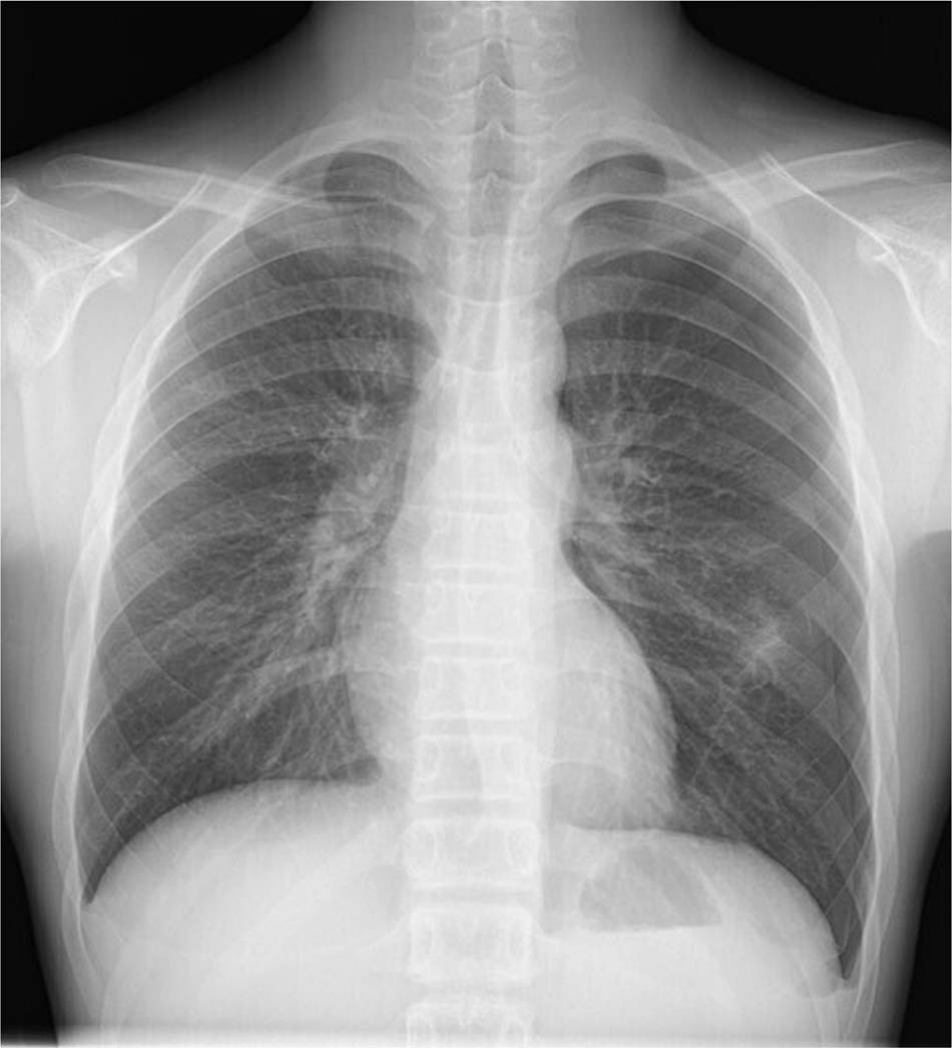
Chest radiography showing a left pneumothorax and an opacity in the left middle lung field.

**Figure 2:**
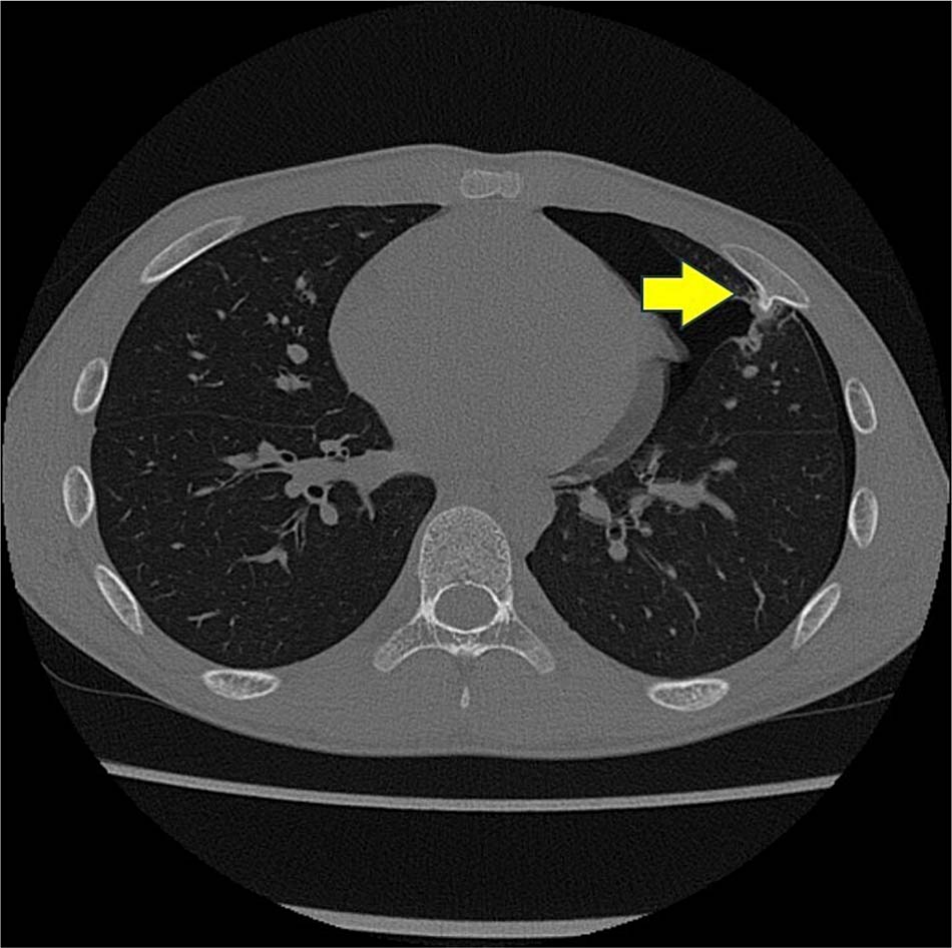
Chest computed tomography scan showing a left pneumothorax and a mass arising from the fifth rib to the thoracic cavity (arrow). The cortical bone and bone marrow continue to move from the fifth rib.

**Figure 3:**
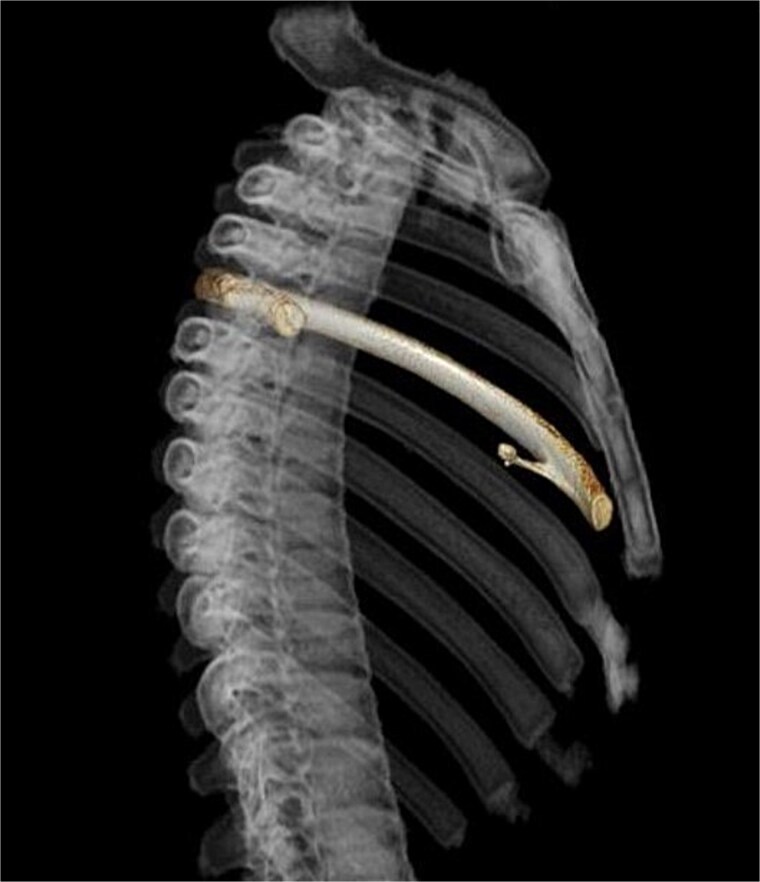
Three-dimensional computed tomography. Slim and pedunculated osteochondroma arising from the anterior fifth rib into the thoracic cavity.

**Figure 4:**
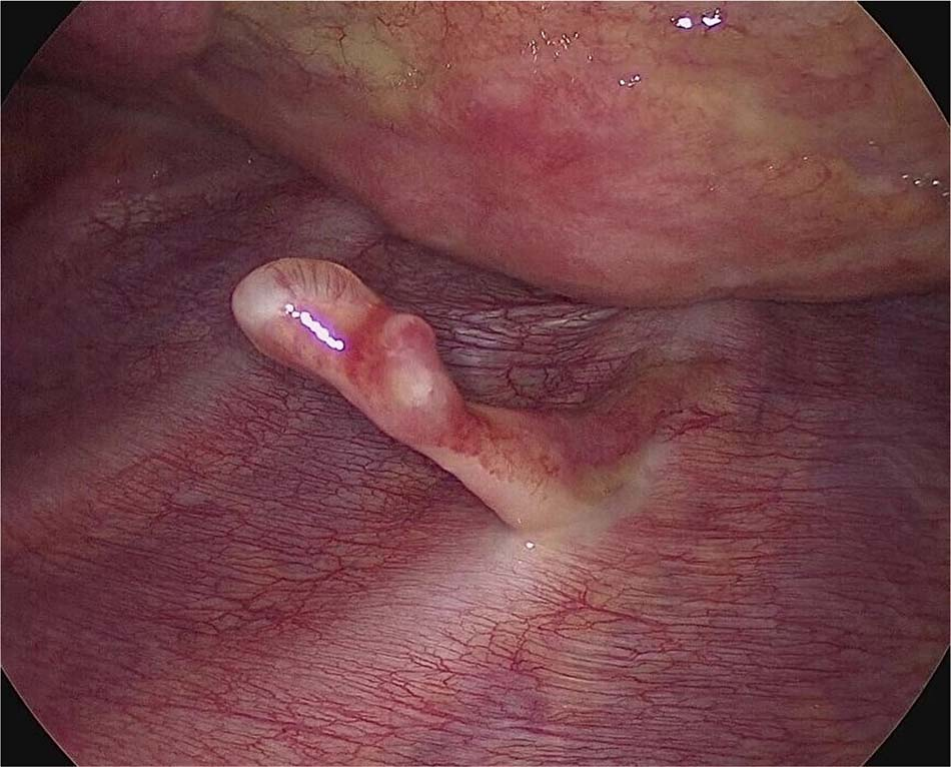
Intraoperative finding of the osteochondroma. Slim and pedunculated osteochondroma arising from the anterior aspect of the fifth rib.

**Figure 5:**
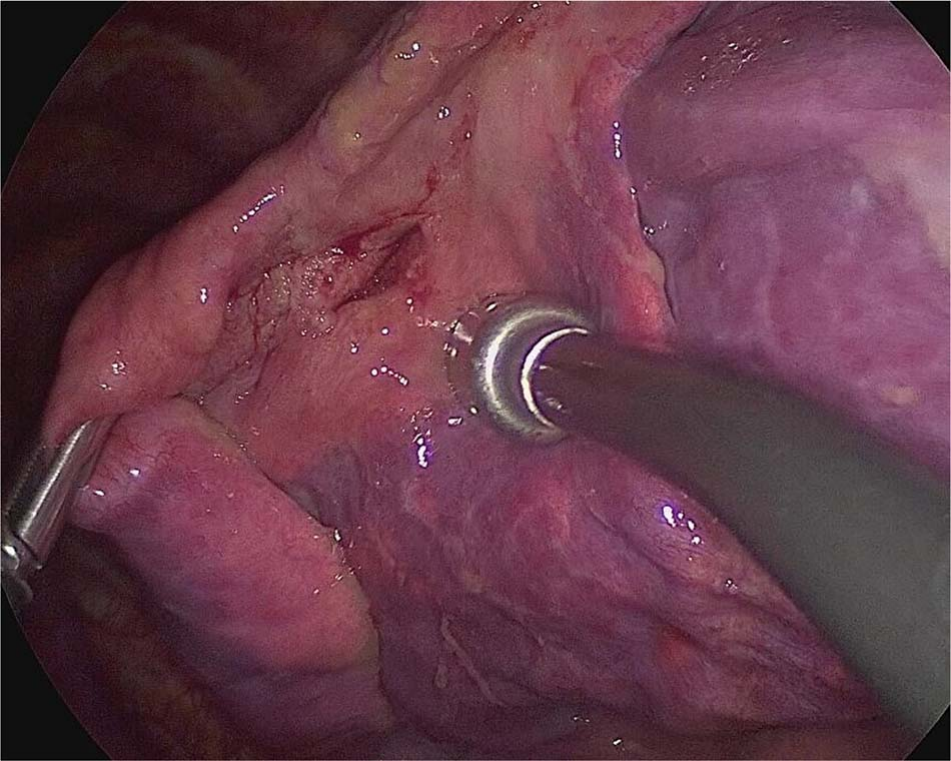
Intraoperative finding of the wound in the left lingular segment. There was a wound in the left lingular segment and a thickened visceral pleura around the wound.

A 13-year-old boy presented with chest pain and dyspnea. There was no significant medical or family history. Chest radiography showed a left pneumothorax and opacity in the left middle lung field (Fig. 1). Computed tomography (CT) showed a left pneumothorax and a bony, pedunculated mass arising from the fifth rib. The cortical bone and bone marrow continued to move away from the fifth rib (Fig. 2). The mass was slim, pedunculated, and pointed directly inward to the thoracic cavity (Fig. 3), and it was suspected to have invaded the left upper lobe of the lung. There were no bullae, and the patient had no history of trauma. The patient was diagnosed with pneumothorax due to a costal osteochondroma. The patient was admitted to our hospital and underwent surgery to remove the costal osteochondroma and treat the left pneumothorax. Three-port video-assisted thoracic surgery revealed no bullae but a bony and pedunculated osteochondroma arising from the anterior aspect of the fifth rib (Fig. 4). There was a wound in the left lingular segment and a thickened visceral pleura around the wound (Fig. 5). We performed wedge resection of the lingular segment, including the wound and thickened visceral pleura. We removed the osteochondroma during thoracoscopic surgery without partial rib resection using electrocautery and Cooper scissors. A water-sealing test revealed no air leakage.

The postoperative course was uneventful. The chest drain was removed on postoperative day 3, and the patient was discharged on postoperative day 4.

Histological examination confirmed the diagnosis of osteochondroma of the fifth rib with complete resection of the cartilage cap and excluded the presence of a malignant lesion. The lung specimen revealed a hemorrhage and thickened visceral pleura around the wound.

We conducted observation postoperatively and there was no recurrence of osteochondroma and pneumothorax.

## Discussion

Osteochondromas generally grow and ossify slowly during skeletal growth and stop growing at skeletal maturity [1, 3]. Osteochondromas are located in the metaphysis and grow away from the joint. Costal osteochondromas typically develop at sites where cartilage is present, such as the costochondral junction and, less frequently, the costovertebral junction [1, 4]. Costal osteochondromas may be difficult to diagnose using chest radiography, and CT is usually suitable [3–5]. One of the key radiological features of osteochondromas on CT is cortical bone and bone marrow continuity between the lesion and parent bone [1]. Patients with osteochondromas are asymptomatic. They become symptomatic due to mechanical compression, injury to adjacent structures, secondary fractures, or even malignant transformations [1, 4]. Costal osteochondromas infrequently cause life-threatening thoracic injuries such as pneumothorax and/or hemothorax because repeated friction between the costal osteochondromas and the visceral pleura due to respiratory motion leads to spontaneous rupture of the visceral pleura and laceration of the lung [4–6]. In the present case, we found a wound and thickened visceral pleura around the wound in the left upper lobe of the lung. These findings revealed that the costal osteochondroma constantly contacted the lungs, which led to lung injury and pneumothorax.

Surgical resection of osteochondromas is recommended when they emerge after puberty, become symptomatic, exhibit rapid growth, or show signs of malignant transformation [4, 7]. Complete resection by removing the osteochondroma at the normal bone base, with consequent removal of the cartilage cap and perichondrium, is recommended [1]. In the absence of remnants of the cartilage cap or perichondrium, the risk of relapse is extremely low [1]. Costal osteochondromas are resected with or without partial rib resection via thoracotomy or thoracoscopic surgery, depending on the case [2–9]. Fujita *et al.* reported that surgical resection via thoracoscopic surgery was effective for slim and pedunculated costal osteochondromas [8]. Three-dimensional CT can clearly describe the morphology and extent of osteochondromas. Thoracoscopy is useful for confirming the location, morphology, and extent of osteochondromas [4]. This may help in determining the surgical approach and procedure. In the present case, the costal osteochondroma was slim and pedunculated in the three-dimensional CT and thoracoscopy. These findings helped us continue and complete the resection of the costal osteochondroma via thoracoscopic surgery without partial rib resection.

In conclusion, osteochondromas are common tumors, whereas costal osteochondromas are rare. We successfully treated a pneumothorax caused by a costal osteochondroma during thoracoscopic surgery.
